# Pneumoscrotum as Complication of Blunt Thoracic Trauma: A Case Report

**DOI:** 10.1155/2013/392869

**Published:** 2013-01-14

**Authors:** Eftychios Lostoridis, Konstantinos Gkagkalidis, Nikolaos Varsamis, Nikolaos Salveridis, Georgios Karageorgiou, Spyridon Kampantais, Paraskevi Tourountzi, Konstantinos Pouggouras

**Affiliations:** ^1^1st Department of Surgery, General Hospital of Kavala, Agios Silas, 65500 Kavala, Greece; ^2^1st Department of Urology, G. Gennimatas General Hospital, Aristotle University of Thessaloniki, 41 Ethnikis Amynis Street, 54635 Thessaloniki, Greece; ^3^1st Department of Surgery, Theagenio Cancer Hospital of Thessaloniki, Alexandrou Simeonidi 2, 54007 Thessaloniki, Greece; ^4^1st Department of Respiratory Medicine, General Hospital of Kavala, Agios Silas, 65500 Kavala, Greece

## Abstract

*Introduction*. Pneumoscrotum is a rare clinical entity. It presents with swollen scrotal sac and sometimes with palpable crepitus. It has many etiologies. One of them is due to blunt trauma of the thoracic cage, causing pneumothorax and/or pneumomediastinum. *Case Presentation*. We report the case of an 82-year-old male who was transferred to the Emergency Department with signs of respiratory distress after a blunt chest trauma. A CT scan was obtained, and bilateral pneumothoraces with four broken ribs were disclosed. Subcutaneous emphysema expanding from the eyelids to the scrotum was observed, and a chest tube was inserted on the right side with immediate improvement of the vital signs of the patient. *Discussion*. Pneumoscrotum has three major etiologies: (a) local introduction of air or infection from gas-producing bacteria, (b) pneumoperitoneum, and (c) air accumulation from lungs, mediastinum, or retroperitoneum. These sources account for most of the cases described in the literature. Treatment should be individualized, and surgical consultation should be obtained in all cases. *Conclusion*. Although pneumoscrotum itself is a benign entity, the process by which air accumulates in the scrotum must be clarified, and treatment must target the primary cause.

## 1. Introduction 

Pneumoscrotum is the general term which describes the presence of air within the scrotum. This term includes scrotal emphysema (or subcutaneous/surgical emphysema of the scrotum) when air is palpated as crepitus and scrotal pneumatocele when air is present within the tunica vaginalis testis but not palpable [1]. The air can derive from three different routes: extraperitoneal sources (retroperitoneum, mediastinum, or lungs), intraperitoneal sources (air-filled hollow viscera), or local sources (gas production or air introduction) [2]. The inciting mechanism must be elucidated, and the appropriate treatment must be provided, especially in life threatening conditions such as pneumothorax or Fournier's gangrene. 

## 2. Case Presentation

An 82-year-old male patient was referred to our accident and Emergency (A & E) Department from a primary level hospital after sustaining a blunt injury of the thorax. The mechanism of the injury was a fall from few stairs without loss of consciousness. On arrival, he had signs of mild respiratory distress (SpO_2_: 92% on room air, RR: 24/min), and he was hemodynamically stable (BP: 157/96 mmHg, HR: 99/min) and alert (Glasgow Coma Scale: 15). During the initial assessment, a subcutaneous emphysema extending from the eyelids to the anterior abdominal wall was palpable. Moreover, the patient had an open wound on his forehead, on the helix of his left ear extending to the ipsilateral external auditory canal, and on his left calf and abrasions at his right forearm. The rest of the physical examination was normal. His past medical history included diabetes mellitus, chronic obstructive pulmonary disease, chronic heart failure, hypertension, dyslipidemia, and hypertension.

Central vein access was gained, and blood tests (complete blood count, complete blood chemistry, blood type, Rhesus determination, and serologic markers for hepatitis) were obtained. By that time, there was no evidence of air inside the scrotum, and a foley catheter was inserted without difficulty. 

Laboratory data revealed elevation of white blood cell count (16.4 K/*μ*L with 91.9% neutrophils), abnormal blood urea nitrogen (68 mg/dL), creatinine level of 2.0 mg/dL, and 20–25 urine RBC per high power field. Other laboratory measurements were unremarkable.

**Figure 1:**
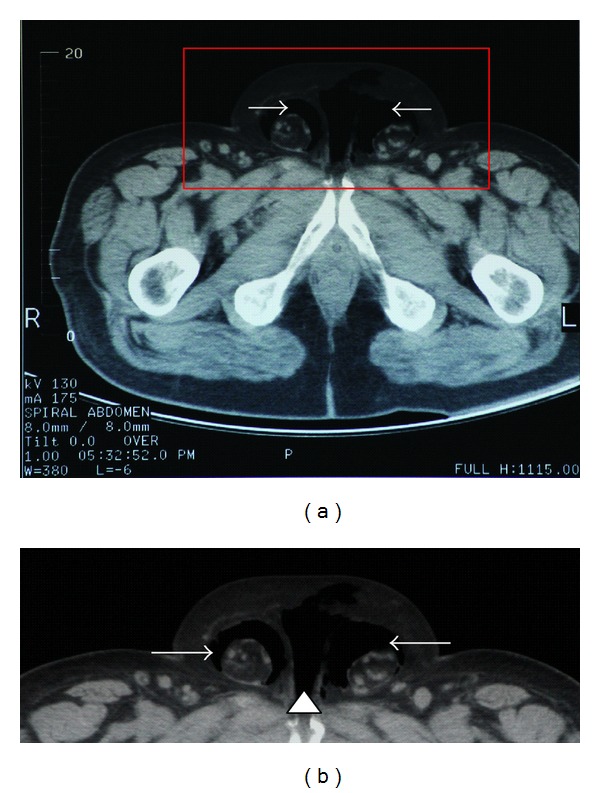
(a) Computed tomography scan of the pelvis. Air (white arrows) is visualized within the scrotal sac (indicative of pneumoscrotum). (b) Magnification of the red frame of (a). Air appears to the scrotum (white arrows) and at the base of the penis (head arrow).

**Figure 2:**
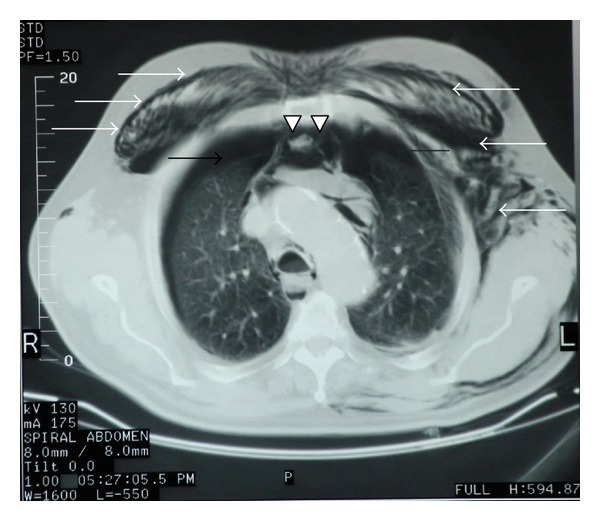
Computed tomography scan of the thorax. Bilateral pneumothorax (black arrows), pneumomediastinum (arrow heads) and subcutaneous emphysema of the anterior and lateral thoracic wall (white arrows).

The patient was transferred to the Radiology Department for basic X-ray studies, ultrasonography exams, and CT scanning (head, cervical spine, thorax, abdomen, and pelvis). The body CT scan revealed a massive subcutaneous emphysema extending from the temporal-parietal region of the head to the scrotum (Figures 1(a) and 1(b)), one broken rib (7th) at the right, and three broken ribs (2nd, 3rd, and 6th) at the left hemithorax, bilateral pneumothorax with pneumomediastinum, and right lower lobe infiltrates (Figure 2). No pneumoretroperitoneum was evident. The rest of the investigations were negative.

**Figure 3:**
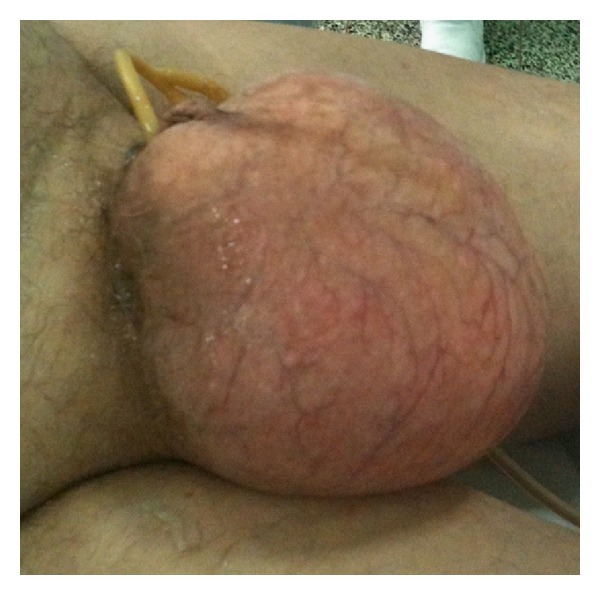
Swelling of the scrotum.

**Figure 4:**
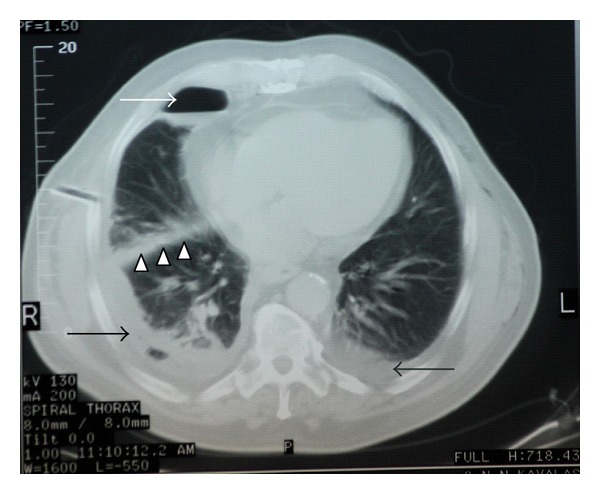
Computed tomography of the thorax at day 13 demonstrates right pneumothorax (white arrow) with lung infiltrations and bilateral pleural effusions (black arrows). The chest tube is visible (head arrows).

During second assessment of the patient, the scrotum had enlarged, and a crepitus was palpable with no septation. A puncture in the scrotum confirmed the diagnosis of pneumoscrotum (Figure 3). A chest tube was inserted to right hemithorax. The left pneumothorax was small (<15% as estimated by the Light's index) and managed conservatively. The rest of the treatment was supportive. Pneumothorax was absorbed after few days of hospitalization. At day 13, he was transferred to the Respiratory Department due to low fever, bilateral pleural effusions, and lung infiltrations, diagnosed by a new chest CT scan (Figure 4). He was treated successfully with parenteral antibiotics for eight days. The rest of his course was uneventful. By the time of discharge, subcutaneous emphysema of the scrotum was completely absorbed. 

## 3. Discussion

Pneumoscrotum is a very rare clinical manifestation. The main symptom is swelling of the scrotum, usually with no pain or tenderness [3]. The first case was described in 1912 following nephrotomy [4]. Since then, few cases have been reported caused either by procedural or pathological processes [1]. Pathologic pneumoscrotum includes pneumothorax (traumatic or spontaneous), pneumomediastinum, visceral perforation, trauma of the abdomen cavity or the retroperitoneum, direct scrotal injury, abscess of the perinephric space, rapid decompression after diving, inflation of the scrotum due to air ejection for sexual purposes, and Fournier's gangrene. Procedural causes of pneumoscrotum derive from liver or kidney biopsy, endoscopy (colonoscopy, sphincterotomy, arthroscopy, peritoneoscopy), laparoscopic procedures [5, 6], retroperitoneal sympathectomy, pacemaker placement, intubation of the trachea, cardiopulmonary resuscitation (CPR), hemorrhoidectomy, chest tube placement, anastomotic leak after large bowel surgery, and artificial pneumoperitoneum for tuberculosis therapy. 

Air accumulation in the scrotum can be explained by three pathophysiologic mechanisms. The first mechanism derives from a gas-producing infection (i.e., Fournier's gangrene) or direct introduction of air (i.e., scrotal trauma). The second mechanism results from the presence of intra-abdominal air. In cases of pneumoperitoneum (usually from visceral perforation), air can reach the abdominal wall (by diffusion or through small peritoneal defects) and can accumulate in the scrotum dissecting along the fascial planes [7]. Sometimes, air can travel to the scrotal sac through a patent processus vaginalis [8]. This embryologic defect is present in 15–30% of adults [9]. The third mechanism includes air spread from a thoracic source (pneumothorax or pneumomediastinum). In the case of pneumothorax, air travels from the lungs along the superficial subcutaneous fascia of Camper's and the deep membranous layer of Scarpa's fascia. These fascial planes are distinct to the abdomen but fuse to form the Colles' fascia at the base of the penis and Dartos fascia in the testes, respectively [1, 10]. In the case of pneumomediastinum, air from the mediastinum travels directly through the diaphragmatic hiatus (periaortic and periesophageal fascial planes) into the perinephric space, causing pneumoretroperitoneum [11]. Then, air expands to the scrotum along the spermatic fascia and the inguinal canal. The mediastinum also communicates with the retroperitoneum through the sternocostal attachment of the diaphragm. This space is continuous with the flanks and the pelvis.

Pneumothorax can cause pneumomediastinum and vice versa. Air from ruptured alveoli travels into perivascular and peribronchial fascial sheath at the lungs roots entering the mediastinum [12]. Less likely, mediastinal pleura may rupture and cause pneumothorax in cases of high pressures or insufficient decompression of pneumomediastinum [13]. 

It must be emphasized that pneumoscrotum itself is a benign condition. Thorough search must be done to find and treat the causative factor of the pneumoscrotum. In case of local infection, proper antibiotics and surgical consultation must be obtained. In case of pneumoperitoneum, the point of air leakage must be clarified and treated accordingly. The treatment of pneumothorax depends on its size and on the patient's condition. A chest tube must be inserted in deteriorating patients. If the cause is pneumomediastinum, conservative treatment is the best choice. Rarely, tension pneumomediastinum must be decompressed immediately. A consultation from a thoracic surgeon must be obtained. A foley catheter must be inserted in order to measure urine output in an unstable patient. Otherwise, it is not mandatory. Urethral compression or vascular compromise is not a concern after pneumoscrotum because the scrotum is elastic and can be distended [1, 2]. 

In our patient, the third pathophysiologic mechanism may apply. The source was from the lungs due to bilateral pneumothorax and pneumomediastinum. The massive subcutaneous emphysema was from air that propagated to the eyelids through the neck and to the scrotum through the abdomen fasciae. Neither pneumoperitoneum nor pneumoretroperitoneum was disclosed by CT scans. 

## 4. Conclusion

We present a rare case of pneumoscrotum after a blunt thoracic injury, diagnosed by CT scan as bilateral pneumothorax and subcutaneous emphysema from the eyelids to the scrotum. There are only few reports of this etiology in the literature. Emergency physicians and surgeons need to be alert when dealing with scrotal swelling and be aware that the primary source of air could be located in the thorax.
